# Evolution and Synthesis of Carbon Dots: From Carbon Dots to Carbonized Polymer Dots

**DOI:** 10.1002/advs.201901316

**Published:** 2019-09-30

**Authors:** Chunlei Xia, Shoujun Zhu, Tanglue Feng, Mingxi Yang, Bai Yang

**Affiliations:** ^1^ State Key Laboratory of Supramolecular Structure and Materials College of Chemistry Jilin University Changchun 130012 P. R. China; ^2^ Laboratory of Molecular Imaging and Nanomedicine National Institute of Biomedical Imaging and Bioengineering National Institutes of Health 35 Convent Dr Bethesda 20892 MD USA; ^3^ State Key Laboratory of Applied Optics Changchun Institute of Optics Fine Mechanics and Physics Chinese Academy of Sciences Changchun 130033 P. R. China

**Keywords:** carbon dots, carbonized polymer dots, formation mechanism, photoluminescence mechanism, synthesis

## Abstract

Despite the various synthesis methods to obtain carbon dots (CDs), the bottom‐up methods are still the most widely administrated route to afford large‐scale and low‐cost synthesis. However, as CDs are developed with increasing reports involved in producing many CDs, the structure and property features have changed enormously compared with the first generation of CDs, raising classification concerns. To this end, a new classification of CDs, named carbonized polymer dots (CPDs), is summarized according to the analysis of structure and property features. Here, CPDs are revealed as an emerging class of CDs with distinctive polymer/carbon hybrid structures and properties. Furthermore, deep insights into the effects of synthesis on the structure/property features of CDs are provided. Herein, the synthesis methods of CDs are also summarized in detail, and the effects of synthesis conditions of the bottom‐up methods in terms of the structures and properties of CPDs are discussed and analyzed comprehensively. Insights into formation process and nucleation mechanism of CPDs are also offered. Finally, a perspective of the future development of CDs is proposed with critical insights into facilitating their potential in various application fields.

## Graphical abstract

**The classification of carbon dots (CDs)** is improved and carbonized polymer dots (CPDs) are revealed with distinctive polymer/carbon hybrid structures and properties, as a new classification of CDs. The synthesis methods of CDs and effects of synthesis on the structures and properties of CPDs are discussed. Furthermore, insights are offered into the nucleation mechanism and the future development of CPDs.

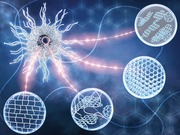

## 1 Introduction

Fluorescent nanomaterials have been extensively employed by scientists owing to their important applications in photoelectric device, biomedicine, etc., such as semiconductor quantum dots,^1^ carbon dots,^2^ polymer dots,^3^ fluorescent nanodiamonds,^4^ and fluorescent nanoclusters.^5^ Especially, the carbon‐based nanomaterials draw increasing attention due to the obvious predominance in respect of environment‐friendly, stability, etc.[qv: 1e,2a,c,4a,6] Carbon dots (CDs), as a new type of fluorescent carbon‐based nanomaterial, have attracted considerable attention since first reported in 2004.[qv: 2d] Owing to their superior optical properties (e.g., strong absorption, bright photoluminescence, excellent light stability, and resistance to light bleaching),^7^ low toxicity,^8^ environment‐friendly,^9^ good biocompatibility,^10^ and facile preparation,[qv: 2a,11] a mass of CDs have been synthesized and they have shown exhilarating advantage in various application fields, including biomedicine,[qv: 10a,12] anticounterfeiting,^13^ sensing,^14^ catalysis,^15^ light‐emitting diode (LED),^16^ and photovoltaic devices,[qv: 9c,17] etc.

The typical CDs are considered as a kind of 0D carbon‐dominated nanomaterial, with the size of less than 20 nm universally, which consisted of sp^2^/sp^3^ carbon skeleton and abundant functional groups/polymer chains.[qv: 7c,18] The large amount of surface groups/polymer chains, such as carboxyl, hydroxyl, amine, etc., gives rise to their excellent water solubility and convenience for compositing with other materials without phase separation.[qv: 7b,19] Moreover, the abundant functional groups make CDs easily modified with various organic or polymeric molecules,[qv: 14a,20] and promising as multifarious sensors. The centric carbon core structure, consisting of sp^2^/sp^3^ carbon atoms, could show graphite lattice or amorphous carbon form,^21^ which can be attributed to the different carbonization degree of CDs. The covalent carbon skeleton structure also enhances the stability of CDs, which is critically important for practical application.[qv: 7b,22] Notably, compared with organic dyes and some traditional quantum dots, containing cadmium or lead, CDs not only have better light stability (resistance to light decomposition, photobleaching, and photoblinking) and high photoluminescence quantum yield, but also possess lower toxicity, lower costs, and excellent biocompatibility.[qv: 2c,23]

Because of multifarious synthesis methods (top‐down and bottom‐up) and wide raw materials (graphite, small molecules, polymers, even natural materials, etc.),[qv: 2a,11,18b] a large family of CDs form up rapidly. In fact, due to the development of synthesis methods and the abundance of raw materials, the CDs have become a universal concept to call the 0D nanomaterial mainly composed of carbon, so CDs were further classified according to the structure and performance features from different systems. Despite extensive debate about the classification and nomenclature of CDs,[qv: 7c,24] it was increasingly clear that CDs could be divided into graphene quantum dots (GQDs), carbon nanodots (CNDs), and polymer dots (PDs) before 2015 in our previous report.[qv: 7c] Cayuela et al. also classified the CDs into GQDs, CNDs, and carbon quantum dots (CQDs).^24^ According to our recent research and summary, we think the CPDs as a kind of new‐form CDs are rising, which possess polymer/carbon hybrid structure rather than the structure of carbon main body.[qv: 7b,10a,13c,22,25] In fact, the CPDs have been widely obtained due to the incomplete carbonization of the polymer clusters by hydrothermal/solvothermal methods, which have been inexactly called CDs before. In this review, we try to improve the classification and nomenclature of CDs, and the CDs are divided into graphene quantum dots, carbon quantum dots, carbon nanodots, and carbonized polymer dots (CPDs) according to the summary and analysis of structure and property features of CDs.

Although a plenty of CDs have been synthesized by kinds of synthesis methods from wide raw materials, it is still difficult yet important to synthesize CDs with predominant performances (strong infrared photoluminescence for bioimaging with strong penetration ability, good electrical conductivity for electroluminescence LED, strong and wide optical absorption for photovoltaic devices, tunable energy structure for good catalytic performance, controllable surface groups for targeting and sensing, etc.).[qv: 9c,14,15,17,23b,25e,26] Besides, many basic scientific issues, such as nucleation and growth process, the influence of synthesis methods and conditions on the structures and properties, morphology and size control, photoluminescence mechanism, etc., have not been well investigated for CDs.[qv: 7c,11,13c,21d,22,27] Especially the development of the simple and highly‐efficient bottom‐up synthesis methods promotes the wide synthesis and investigation of CDs, which also make it difficult to give out the consistency structure and photoluminescence mechanism in different systems. So, it is significant to reveal and establish the linkages between synthesis and properties. Herein, we summarized all kinds of synthesis methods for the preparation of CDs, especially focused on the CPDs and relevant synthesis methods (hydrothermal, solvothermal, microwave‐assisted method, etc.). Not only the structure and properties of CPDs are discussed in detail, but also the effects of synthesis conditions on the structure and properties are analyzed according to the current published reports. The nucleation mechanism and forming process of CPDs in hydrothermal condition, which are always unclear, are also major topics to discuss. Besides, some opinions and challenges are also suggested to bring valuable insight into the future development needs of CPDs and CDs.

## 2 Classification of CDs

**Figure 1:**
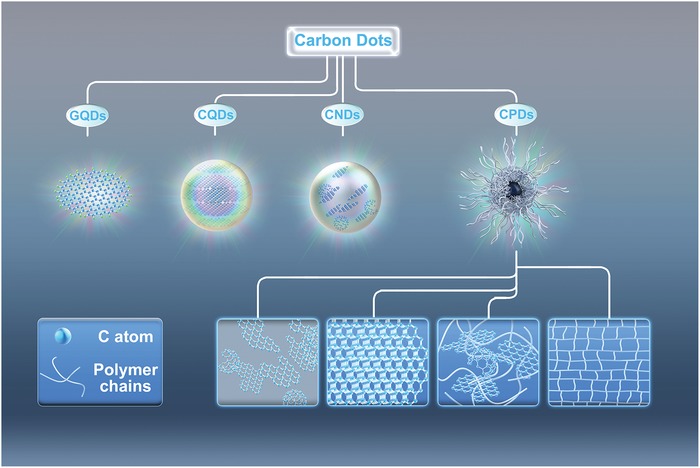
Classification of CDs: including graphene quantum dots (GQDs), carbon quantum dots (CQDs), carbon nanodots (CNDs), and carbonized polymer dots (CPDs), and the possible structures of carbon core of CPDs.

CDs, as the generic term for a variety of nanosized fluorescent carbon materials, concretely include graphene quantum dots, carbon quantum dots, carbon nanodots, and carbonized polymer dots, which are classified according to the specific carbon core structure, surface groups, and properties, as is shown in **Figure** **1**.[qv: 7c,13c,24,25d] The GQDs are small graphene fragments consisting of single or few graphene sheets with obvious graphene lattices and chemical groups on the edge or within the interlayer defect, which contribute the unique properties, such as quantum confinement effect and edge effect.[qv: 14b,28] They are typically anisotropic with lateral dimensions usually less than ≈20 nm and height usually less than five layers graphene sheets (≈2.5 nm).[qv: 14b,18a,29] It is worthy to mention that the quantum confinement effect not only indicates the quantum confinement effect of GQDs' sizes, it also refers to the quantum confinement effect of the conjugated π‐domains, which are isolated by the defects on the plane of graphene. Usually, the increasing of the oxygen element would increase the number of defects resulting in the more conjugated π‐domains as the fluorescence centers.[qv: 7c,27a,30] The CQDs are always spherical and possess obvious crystal lattices and chemical groups on the surface, which show intrinsic state luminescence and quantum confinement effect of the CQDs' size. It makes the significant meaning to regulate the wavelength of photoluminescence through the tune of the CQDs' size.[qv: 21a,31] The CNDs possess high carbonization degree with some chemical groups on the surface, but usually show no obvious crystal lattices structure and polymer features, and the photoluminescence mainly originates from the defect/surface state and subdomain state within the graphitic carbon core without quantum confinement effect of the particle size.[qv: 27a,32] The CPDs possess a polymer/carbon hybrid structure comprising of abundant functional groups/polymer chains on the surface and a carbon core. The carbon core can be divided into four subclasses: two kinds of completely carbonization cores similar to the CNDs or CQDs,^33^ a paracrystalline carbon structure composed of tiny carbon clusters surrounded by polymer frames,[qv: 32a,34] and a highly dehydrated crosslinking and close‐knit polymer frame structure.[qv: 13c,35] For CPDs, the photoluminescence mainly originates from the surface state, subdomain state, molecular state, and crosslink enhanced emission (CEE) effect. Especially, the molecular state and CEE effect contribute the predominant optical property, and the other kinds of CDs do not have these photoluminescence features and mechanism.[qv: 13c,22,25b,d,32a,36]

## 3 Structural and Optical Properties of CPDs

As GQDs, CQDs, and CNDs have been widely reported in a lot of reviews,[qv: 2a,11,14b,23b,28b] to avoid repetition we would only focus on the structural and optical properties of CPDs in this section.

### 3.1 Structural Properties of CPDs

The CPDs show a polymer/carbon hybrid structure rather than the structure of carbon main body, which contribute the predominant properties of the CPDs, different from neither the traditional carbonized CDs (GQDs, CQDs, and CNDs) nor the PDs. The CPDs not only possess the prominent optical properties of the traditional carbonized CDs but also inherit the property of polymer.[qv: 18b,25d] They have unique characters such as high oxygen/nitrogen content, excellent aqueous solubility, and outstanding photoluminescence quantum yield (PLQY), which are attributed to the polymer/carbon hybrid structure and special photoluminescence mechanism.[qv: 7b,13c,21c,e,25d,35] The polymeric properties mainly include three aspects: abundant functional groups and short polymer chains, which are reserved owing to incomplete carbonization; polydispersity in structures and properties influenced by complex polymerization process and inexact reaction conditions; and highly crosslinked network structure generated by the process of dehydration and carbonization.[qv: 13c,18b,35] The polymeric properties are not found in the GQDs, CQDs, and CNDs. It should be noted the CPDs are distinguished from the polymer dots, which lack carbonization or highly crosslinked and close‐knit polymer frame structure.[qv: 3b,37] As a result, the CPDs can be regarded as a transition material between PDs and fully carbonized CDs, which might be also a new material.[qv: 25d]

**Figure 2:**
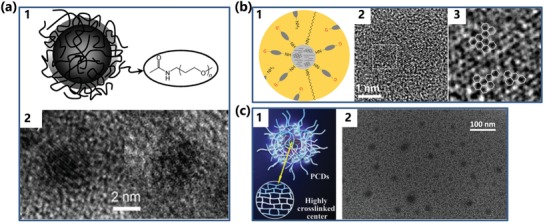
Structures of the carbon core: a) CPDs with the complete carbonization core: 1) representation of a CPD containing an oligomeric PEG diamine on the surface; 2) the high resolution transmission electron microscope (HRTEM) images of two CPDs with obvious crystal structure and amorphous carbon structure, respectively. a) Reproduced with permission.[qv: 33a] Copyright 2010, Wiley‐VCH. b) CPDs with a paracrystalline carbon structure core: 1) representation of a CPD; 2) the HRTEM image of the CPDs; the area surrounded by the dotted square in (2) is magnified in (3). b) Reproduced with permission.[qv: 34a] Copyright 2015, Nature Publishing Group. c) CPDs with a highly dehydrated crosslinking and close‐knit polymer frame structure core: 1) representation of a CPD; 2) the TEM image of the CPDs. c) Reproduced with permission.^35^ Copyright 2017, Elsevier.

In our opinion, the CPDs should usually be obtained through the carbonization of polymer clusters, so the carbonization degree, as an important parameter to evaluate the polymer/carbon hybrid structure of CPDs, has an important effect on the properties of CPDs.[qv: 13c,22,32a] Besides, the CPDs can also be reached by the postdecoration of the CNDs and CQDs with polymer or organic molecules.[qv: 33a,38] The CPDs prepared by postdecoration possess the complete core structures of CQDs or CNDs with a clear boundary between the core and polymer shell. On the contrary, the CPDs prepared by the carbonization of bottom‐up methods usually exist with no clear boundary between the core and shell. The carbon core of CPDs is supported to be the complete carbonization core, concretely similar with two kinds of core structures of CQDs or CNDs; the cores can show intrinsic state or subdomain state emission, respectively (**Figure** **2**a),[qv: 33a,b] or a paracrystalline carbon core structure composed of tiny carbon clusters surrounded by polymer frames (Figure 2b),[qv: 32a,34] or a partly dehydrated crosslinking and close‐knit polymer structure core which could form an ordered structure and can be as steady as completely carbonized carbon skeleton (Figure 2c).[qv: 13c,21c,35] The tiny carbon clusters as the subdomain in the carbon core could be not only the conjugated π‐structure but also the diamond‐like structure, and the conjugated π‐structure could be planar or curved, such as fullerene fragments or fullerene structure, etc.[qv: 32a] Collectively, these reports indicate that some CPDs show amorphous structure, while some other show the lattice structure under the transmission electron microscope (TEM). The observed crystal lattice structure could be not only the lattice of graphite but also ordered and compact polymer frame structure.[qv: 18b,39] That is the reason why the lattice spaces of many reported CDs are inconsistent with graphite lattice spaces. Besides, the graphite lattice spaces could occur with variation due to the polymer/carbon hybrid structure, such as the polymer chain is inserted between graphite structures resulting in the variation of lattice spaces.[qv: 28c]

### 3.2 Optical Properties of CPDs

#### 3.2.1 Light Absorption of CPDs

**Figure 3:**
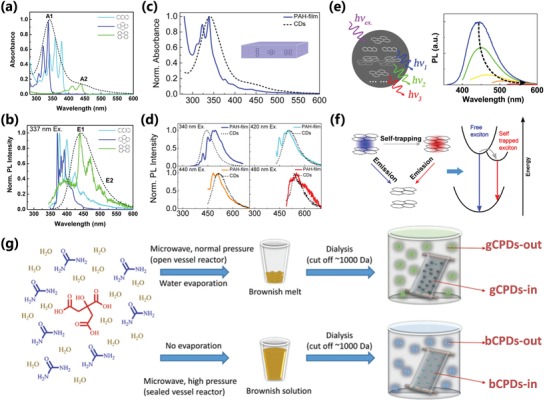
a) Absorption spectra of the films prepared from anthracene (light blue), pyrene (dark blue), perylene (green) dispersed in PMMA matrix at 0.01% molar concentration, and absorption spectra of the CPDs aqueous solution (black dashed line). b) Normalized photoluminescence spectra of the corresponding films in (a) excited at 337 nm. c) Normalized absorption of a blend of polycyclic aromatic hydrocarbons (blue) with a molecular ratio of 10:10:1:20 anthracene/pyrene/perylene/PMMA monomer unit in PMMA film and the CPDs aqueous solution (black dashed line). d) Photoluminescence spectra of the same film in (c) excited at different wavelengths and the photoluminescence spectra of the CPDs (black dashed line) as a reference. e) The schematic diagram of fluorescence emission and origin of the CPDs. f) The schematic illustration of the exciton self‐trapping process in a pyrene molecule pair: a free exciton (blue spot) may be self‐trapped in a molecule pair as a self‐trapped exciton (red spot), resulting in the reduction of energy and mobility of the exciton, the absorption and emission are influenced by the interaction between fluorophores. a–f) Reproduced with permission.[qv: 40a] Copyright 2015, American Chemical Society. g) The schematic illustration of the synthesis and purification steps of bCPDs (blue emission) and gCPDs (green emission), the fluorophores of molecular state run out of the dialysis bag and get out of the CPDs' nanoparticles. Reproduced with permission.^43^ Copyright 2018, The Royal Society of Chemistry.

CPDs usually exhibit apparent light absorption in the UV–vis region, especially showing a strong absorption in the UV region.[qv: 2a,7c] According to the structural properties, the polydisperse and complex polymer/carbon hybrid structure, the absorption of CPDs is a comprehensive result from multiple structures in the CPD nanoparticles, which can be seen as a fluorescent cocktail or nanoclusters containing complex ingredients.^40^ Generally, the maximum absorption peak at ≈230 nm is attributed to π–π* transition from aromatic carbon structure,[qv: 7c,34a,40a] while the absorption shoulder peak at ≈300 nm is ascribed to n–π* transition from functional groups with lone pairs electron, including the amino‐based chromophores, etc.[qv: 25b,41] The molecular state–related structures possessing the bandgap similar to the organic fluorescent molecules would also generate characteristic absorption peaks.^22^ The absorption in the longer wavelength region is assigned to the energy level transition from the angstrom‐sized conjugated π‐structure.[qv: 25e,32a,34,40a] The derivatives of above structure connected with various chemical groups (such as hydroxyl and amine, etc.) could alter the absorption features of CPDs.^11^, ^42^ Besides, the compact structure within the CPDs enhances the interactions among groups, which make the energy gap change, resulting in variation of the absorption (**Figure** **3**a,c,f).[qv: 25d,40a,42a]

#### 3.2.2 Photoluminescence of CPDs

The photoluminescence of CPDs is one of the most valuable properties as a research focus. The reported CPDs mostly possess strong blue and green emission, even several long‐wavelength emissive CPDs are recently reported.[qv: 2d,7b,25e,27d,44] The majority of the CPDs usually possess the excitation‐dependent emission, which come from different fluorescence centers and complex energy levels.[qv: 32a,40,45] Because each one of the CPD nanoparticles contain plentiful fluorophores as a fluorescent cocktail, the CPDs have a wide composition distribution, and each fluorophore may be in different chemical environment. The chemical environment can lead to the change of photoluminescence property, such as the environment with different polarity results in the shift of emission wavelength just as the solvent‐dependent emission. Diverse photoluminescence states in the CPDs exist resulting in excitation‐dependent emission (Figure 3b,d–f).[qv: 32a,40] The excitation‐dependent emission as a unique feature was utilized to tune photoluminescent color by thickness‐dependent or concentration‐dependent methods in film or solution.[qv: 41a,46] The full width at half maximum (FWHM) of the CPDs is generally larger than semiconductor quantum dots, and the larger FWHM of the CPDs can also be attributed to the polydispersity of fluorescence centers due to the same reasons of the excitation‐dependent emission. So, under a excitation wavelength, multiple energy level transitions from polydisperse fluorescence centers can occur resulting in the emission possessing a wide wavelength range with a large FWHM.[qv: 13c,16b,40a] The CPDs generally have higher quantum yield than completely carbonized CDs (GQDs, CQDs, and CNDs) owing to the special photoluminescence mechanism, for example, the molecular state emission, crosslink enhanced emission effect, and the surface state emission from the surface passivation of the polymer/carbon hybrid structure, etc.[qv: 13c,22,25b,33a,43,45b,47] Song et al. reported that the IPCA molecule contributes to the main emission of the CPDs prepared from citric acid and ethanediamine. The molecular state converting to carbon core state generally results in the degressive PLQY.[qv: 7b,22] In fact, organic molecules of molecular state are easy to get out of the nanoparticles of CPDs resulting in the decreasing of fluorescence of CPDs. However, when the small molecules are confined in the skeleton of polymer/carbon hybrid by the covalent bonds, supramolecular interactions, the space‐limited interactions, etc., as a molecular state related structure, the fluorescence of molecular state is thus truly from the nanoparticles of CPDs rather than the unbonded organic fluorescent molecules (Figure 3g).[qv: 13c,22,43] The molecular state–related structure incorporated in the polymer/carbon hybrid skeleton of the nanoparticles can make the fluorescence property show more advantages than the organic fluorescent molecules in some respects, such as better stability and lower toxicity for biological application. Sun's group and Peng's group reported the carbon nanoparticles were passivated by modifying polymer or organic molecules on the surface to form CPDs, which dramatically increased the photoluminescence.[qv: 33a,38a,c,48] The photoluminescence of the CPDs prepared by postdecoration usually originates from the surface state luminescence due to the passivation effect. It also supports that the incompletely carbonized CPDs, prepared by carbonization of polymer clusters, form the self‐passivated structure, which is favorable for the stronger photoluminescence.[qv: 27c] Zhang's group reported that the incompletely carbonized CPDs have higher PLQY than the oxidized GQD obtained under higher temperature.[qv: 21e] The reasons of stronger photoluminescence can be attributed to the surface passivation and CEE effect.[qv: 7c,25d,45b] Besides, some CPDs have the nature of up‐conversion photoluminescence,[qv: 21e,25e,38b,49] pH and solvent dependent photoluminescence, etc.[qv: 10a,14a,18b] However, as the CPDs inherit the polymeric properties and their photoluminescence probably come from molecular state–related structure and subfluorophores, the CPDs usually do not show the quantum size effect, even the stability against photobleaching, etc., which is different from the CQDs or CNDs.[qv: 7b,25d,45b]

#### 3.2.3 Photoluminescence Mechanism of CPDs

**Figure 4:**
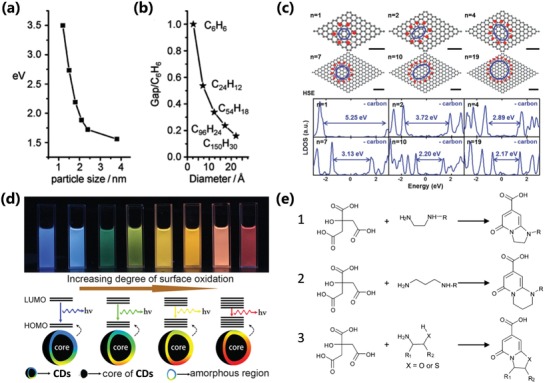
a) The variation of photoluminescence properties with the sizes of CQDs; b) HOMO–LUMO gap dependence on the size of the graphite fragments. a,b) Reproduced with permission.[qv: 21a] Copyright 2010, Wiley‐VCH. c) Schematic representation of the calculated atomic structures and their local density of states using Heyd–Scuseria–Ernzerhof (HSE) hybrid functional with different number of sp^2^ carbon ring confined by surrounding hydroxyl groups, which serve as subdomain to create the bandgap resulting in photoluminescence (gray, C atoms; red, O atoms; white, H atoms; and purple, K atoms). c) Reproduced with permission.[qv: 27a] Copyright 2016, Wiley‐VCH. d) Schematic illustration for the tunable photoluminescence of CPDs by changing the surface state with different degrees of surface oxidation. d) Reproduced with permission.[qv: 36c] Copyright 2015, American Chemical Society. e) The potential molecular state fluorophores in CA‐based CPDs prepared from different nitrogen‐containing precursors, the R can be small molecular groups or polymer chains. e) Reproduced with permission.^22^ Copyright 2015, The Royal Society of Chemistry.

**Figure 5:**
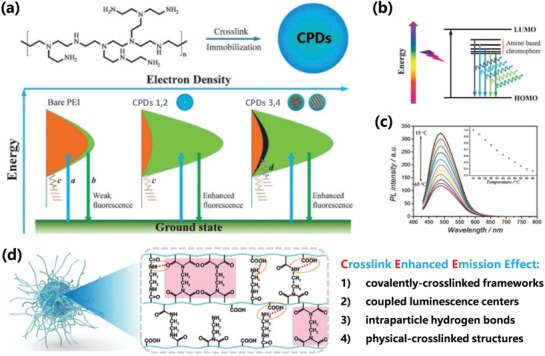
a) Schematic representation for the photoluminescence mechanism (CEE effect) of bare PEI and CPDs 1–4. a: Electrons excited from the ground state and trapped by the amino‐based states; b: excited electrons return to the ground state via the radiative route; c: excited electrons return to the ground state via a vibration and rotation nonradiative route; d: excited electrons return to the ground state via a carbon core‐based nonradiative route. b) The suggested excited level for CPDs with excitation‐dependent behaviors. c) The temperature‐dependent photoluminescence of CPDs. a–c) Reproduced with permission.[qv: 25b] Copyright 2014, The Royal Society of Chemistry. d) Schematic illustration of CEE effect with specific crosslinking forms. d) Reproduced with permission.[qv: 25a] Copyright 2018, Wiley‐VCH.

Photoluminescence mechanism of CDs have been violently controverted and four photoluminescence mechanism are universally accepted: carbon core state luminescence from conjugated π‐domain with quantum confinement effect, surface state luminescence from the interaction of surface groups and carbon core, molecular state luminescence from the specific molecular structure that is similar with organic luminescent molecules, and crosslink enhanced emission effect.[qv: 7c,22,28e,38c,50] The aforementioned photoluminescence mechanisms were applied to explain the luminescence of various types of CDs, and they need to be comprehensively analyzed and combined with the latest investigations of CPDs to give out reasonable photoluminescence mechanism of CPDs. The carbon core luminescence could derive from the intrinsic emission of carbon core with the perfect graphite structure, and the bandgap can be regulated by the size of carbon core.[qv: 7c,16b,26a] However, because the CPDs usually should be incompletely conjugated, sp^2^/sp^3^ hybrid carbon skeleton structure, which is different from CQDs, mismatches the direct relationship between size and luminescence.[qv: 13c,27a,b,32a,51] But the luminescence of CPDs can also be influenced by the size of conjugated π‐domain, which is considered as a subdomain as fluorescence center within CPDs called as subdomain state. So, the carbon core luminescence can also derive from the subdomain emission from the conjugated π‐domain within the graphitic carbon core. The subdomain possesses smaller size than the exciton Bohr radius, so the principle of the quantum confinement effect should be likewise suitable for the luminescent subdomain, which can be considered as a kind of subdomain conducted quantum confinement effect. So, the red‐shift emission as the increasing carbonization degree can be attributed to the growth of subdomain even though the sizes are invariable, which is different from the CQDs (**Figure** **4**a–c).[qv: 6b,21a,27a,32a,34,40a,52] The surface state luminescence is an important origin of fluorescence for CPDs owing to the special polymer/carbon hybrid structure of a carbon core with plenty of polymer chains on the surface. In some cases, the polymer or organic molecules passivated surface dominated the optical properties of the CPDs due to the passivation of surface defect sites and formation of surface energy level. The passivation of surface defect sites reduces the quenching of the exciton caused by the surface trap resulting in enhanced fluorescence. The photoluminescence of surface state was also tunable by regulating the surface chemical structures to change the surface energy level (Figure 4d).[qv: 21b,27c,33a,36c,38b,c,48] Besides, the molecular state luminescence and CEE effect as the important photoluminescent mechanism contribute to only the photoluminescence of the CPDs, which have no contribution for GQDs, CQDs, and CNDs. The CPDs may have some specific molecular structure incorporated in the polymer/carbon hybrid skeleton, which serve as fluorescence centers to contribute the luminescence just as the organic fluorescent molecules (Figure 4e), and the molecular state luminescence can be enhanced by the CEE effect, even the subfluorophores without fluorescence in isolation can produce fluorescence by the CEE effect. It is the reason that the crosslinking can restrict the vibration and rotation to reduce the nonradiation relaxation de‐excitation process, resulting in increasing the radiative transition probability to contribute to enhanced fluorescence (**Figure** **5**a,b). Similarly, lower temperature can also restrict the vibration and rotation leading to stronger photoluminescence (Figure 5c). The crosslinking specifically includes covalently crosslinked frameworks, coupled luminescence centers, intraparticle hydrogen bonds, and physical‐crosslinked structures, etc. (Figure 5d).[qv: 13c,22,25a,b,45b,47,53] It should be noted that the carbonization prompts the forming of the rigid structure, which sequential plays a leading role in physical crosslinking points to create the CEE effect to enhance the emission. As a consequence, the photoluminescence can be enhanced through the increasing carbonization degree,[qv: 8b,9d,13c,21e,54] and it has been pointed out as a specific carbonation‐induced CEE effect by Xia et al.[qv: 13c]

## 4 Synthesis Methods of CDs

### 4.1 Top‐Down Methods

**Figure 6:**
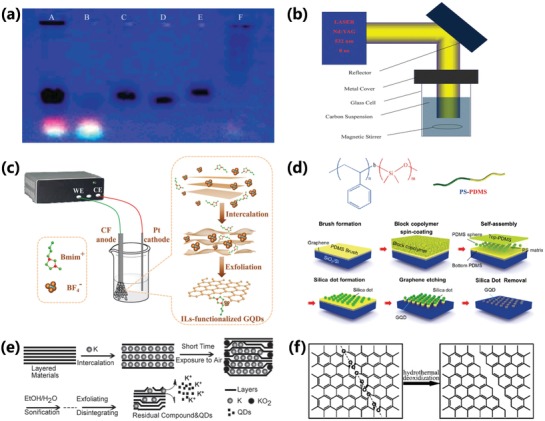
a) Electrophoretic profile under 365 nm UV light for separating A) crude SWNTs suspension; B) fluorescent carbon; C) short tubular carbon; D,E) further separation of (C); F) cut SWNTs. a) Reproduced with permission.[qv: 2d] Copyright 2004, American Chemical Society. b) A schematic illustration of experimental setup for laser‐ablation method. b) Reproduced with permission.[qv: 56a] Copyright 2011, The Royal Society of Chemistry. c) Schematic illustration of the electrochemical exfoliation of carbon fibers in pure ionic liquid electrolyte for the preparation of CDs. c) Reproduced with permission.[qv: 57d] Copyright 2017, The Royal Society of Chemistry. d) Chemical structure of the PS‐PDMS BCP and schematic illustration of the fabrication of GQDs including the spin‐coating of BCP, formation of silica dots, and etching process by O2 plasma. d) Reproduced with permission.[qv: 30a] Copyright 2012, American Chemical Society. e) The schematic representation of the formation of GQDs by intercalating K atoms between the layered graphene sheets. e) Reproduced with permission.[qv: 60c] Copyright 2012, The Royal Society of Chemistry. f) The schematic representation of the formation mechanism of GQDs by hydrothermal cutting of oxidized GSs. f) Reproduced with permission.[qv: 28c] Copyright 2010, Wiley‐VCH.

Usually, CDs are formed by cutting larger carbon structure materials during top‐down synthesis process. The top‐down method mainly includes arc discharge,[qv: 2d,55] laser ablation,[qv: 31a,33a,38b,c,48,56] electrochemical synthesis,[qv: 21a,57] nanometer etching,[qv: 28f,30a] and hydrothermal/solvothermal/special oxidation cleavage,[qv: 10b,28c–e,58] etc. In 2004, Xu et al. discovered a mixture of unknown fluorescent nanoparticles when separating the single‐walled carbon nanotubes (SWNTs) from the arc discharge soot, and it marks the first discovery of CDs (**Figure** **6**a).[qv: 2d] In 2006, CDs were synthesized via a laser ablation method by Sun's group, in which, a carbon target, prepared by hot‐pressing a mixture of graphite powder and cement, was ablated using a Q‐switched Nd:YAG laser (1064 nm, 10 Hz). The obtained carbon nanoparticles have no detectable fluorescence and further passivated by polymer to reach bright luminescence. After that, CDs prepared by laser ablation method were reported widely, which included laser‐ablation of graphite in different solvents, etc. (Figure 6b).[qv: 31a,33a,48,56] Hu et al. synthesized CDs by laser ablation of a suspension of carbon powders in PEG_200N_ solvent (PEG: poly(ethylene glycol)), which simultaneously serve as surface passivation agent. The obtained CDs with a lot of polymer chains on the surface have strong blue emission.[qv: 31a] Electrochemical synthesis of CDs has attracted great interest since first reported by Zhou et al.[qv: 57a,b,g] Due to the simple operation and readily available equipment, the electrochemical synthesis method was used more widely than arc discharge and laser ablation methods. Usually, the applied voltage and electrochemical reaction at the electrode lead the carbon electrode materials to be corroded and exfoliated to reach CDs (Figure 6c). Several kinds of carbon electrode materials and electrolytes were used, including graphite rods, multiwalled carbon nanotubes (MWCNTs), carbon fibers, graphite powder, etc., as the carbon source, and sodium hydroxide, 1‐butyl‐3‐methylimidazolium tetrafluoroborate ([BMim][BF4]), tetrabutylammonium perchlorate, phosphate buffer solution, potassium persulfate, even ultrapure water, etc., as the electrolytes.[qv: 14a,21a,26b,57,59] Nanometer etching strategy, as a strong physical means, was also used for the preparation of CDs.[qv: 28f,30a] Lee et al. utilized the self‐assembly of Si‐containing polystyrene‐bpolydimethylsiloxane (PS‐PDMS) block copolymers (BCPs) to achieve the fabrication of uniform and controllable sized GQDs (Figure 6d).[qv: 30a] The researches of alkali metal–graphite intercalation compounds also provided an avenue to prepared CDs.^60^ Lin et al. utilized the metal–graphite intercalation method to exfoliate and disintegrate the multiwalled carbon nanotubes or graphite flakes (GFs) to reach water‐soluble CDs, which possessed obvious graphite structure (Figure 6e).[qv: 60c] Hydrothermal/solvothermal/special oxidation cleavages have also been widely reported for preparation of CDs due to the simple operation.[qv: 28c,e,58a–d] The hydrothermal strategy, as a chemical route to cut graphene sheets (GSs) into surface‐functionalized CDs, was first reported by Wu's group (Figure 6f).[qv: 28c] Zhu et al. reported a one‐step solvothermal route to obtain CDs, in which the graphene oxide (GO) was dissolved in DMF and pyrolyzed in a poly(tetrafluoroethylene) (Teflon)‐lined autoclave.[qv: 58a] Besides, acid oxidation[qv: 28e,58e] and photo‐Fenton reaction[qv: 28d] are also utilized to synthesize CDs by oxidation cleavage.

### 4.2 Bottom‐Up Methods

**Figure 7:**
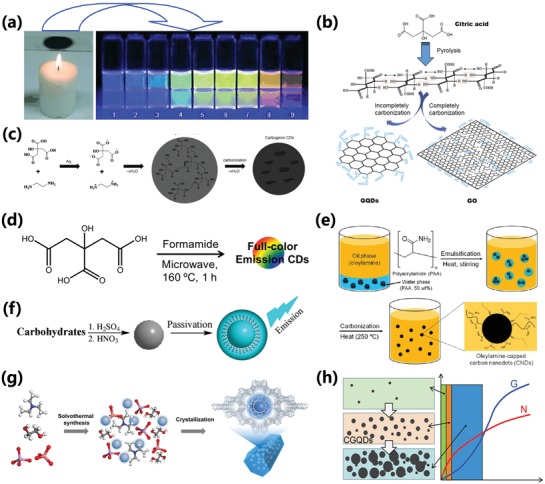
a) Multicolor fluorescent CDs obtained from the combustion soot of candles. a) Reproduced with permission.[qv: 61a] Copyright 2007, Wiley‐VCH. b) Schematic representation of synthesis of GQDs and GO from the pyrolysis of citric acid. b) Reproduced with permission.^66^ Copyright 2012, Elsevier. c) Synthetic route of using citric acid and ethylenediamine to prepare CDs: from ionization to condensation, polymerization, and carbonization. c) Reproduced with permission.[qv: 7b] Copyright 2013, Wiley‐VCH. d) Schematic representation of full‐color emission CDs prepared from microwave‐assistant pyrolysis. d) Reproduced with permission.[qv: 65b] Copyright 2015, Wiley‐VCH. e) Schematic representation of formation of oleylamine‐capped CDs from carbonization of polyacrylamide in oleylamine emulsion micelles. e) Reproduced with permission.[qv: 73a] Copyright 2013, American Chemical Society. f) Schematic representation of dehydration and carbonization of carbohydrate caused by concentrated sulfuric acid to prepared CDs. f) Reproduced with permission.[qv: 38a] Copyright 2009, American Chemical Society. g) Schematic representation situ formation process of photoluminescent CDs@zeolites composite. g) Reproduced with permission.[qv: 75b] Copyright 2016, AAAS. h) The effect of nucleation rate and growth rate for the CVD synthesis of GQDs. h) Reproduced with permission.^76^ Copyright 2013, Wiley‐VCH.

On the other hand, the bottom‐up method includes combustion,[qv: 32b,61] pyrolyzation,[qv: 8a,31c,62] hydrothermal,[qv: 7b,13c,31d,63] solvothermal,[qv: 7a,33b,34a,64] and microwave assistant pyrolysis,[qv: 21e,46b,65] etc., in which CDs are usually synthesized by molecular or/and polymeric precursors. The combustion soot of candles or natural gas burners have been reported to synthesize CDs.[qv: 32b,61] Liu et al. first reported the preparation of CDs by oxidizing the combustion soot of candles in the HNO_3_, followed by purification and separation utilizing polyacrylamide gel electrophoresis (PAGE), resulting in photoluminescent CDs with different emitting wavelength ranging from 415 to 615 nm (**Figure** **7**a).[qv: 61a] Milder than combustion, one‐step pyrolysis method by high‐temperature treatment of the small molecular precursors was also used for preparation of CDs.[qv: 8a,26c,31c,32a,62,66] Chi and co‐workers prepared the GQDs by incompletely pyrolyzing citric acid, and the complete carbonization of citric acid resulted in preparation of graphene oxide (Figure 7b).^66^ Hydrothermal, as an economic, convenient, environment‐friendly synthesis method, is widely popular with researchers, which was widely used for preparation of CDs from kinds of raw materials, such as natural products,[qv: 9a,c–e,67] polymer,[qv: 25c,27b,35] and small molecules,[qv: 7b,47a,68] etc. The obtained CDs by hydrothermal routes not only have prominent optical properties but also the post‐treatments are generally simple. In 2013, Zhu et al. successfully prepared the highly blue emissive CDs with an ≈80% PLQY, which was considered a significant advance in the field of CDs (Figure 7c).[qv: 7b,23b] Similar to the hydrothermal routes, solvothermal routes were also used widely for preparation of CDs.[qv: 7a,19a,33b,64,65,69] Several kinds of solvent were used, such as ethanol, DMF, glycol, formamide, etc. The polarity of solvents has great and important influences on the optical properties of CDs by regulating the carbonization process.[qv: 33b,64,68] So, the solvent‐engineered strategy was used to regulate the properties of CDs.[qv: 27d,70] Ding et al. obtained CDs with different emission wavelengths by the solvent‐engineered strategy. They found different solvent have an influence on the dehydration and carbonization processes resulting in modulating the graphitic nitrogen content and sizes of CDs.[qv: 27d] Microwave as an excellent source of energy could increase the rate of reaction, so utilizing microwave‐assistant approach to synthesize CDs has the fast and high‐efficiency characteristics, which makes this synthesis method to be used widely for preparation of CDs.[qv: 21e,46b,65,71] Pan et al. heated the citric acid formamide solution by microwave‐assistant approach to fleetly synthesize truly fluorescent excitation‐dependent CDs with full‐color emission (Figure 7d). Not only the microwave‐assistant approach, but also the ultrasonic wave as a kind of energy source was used to prepare CDs.[qv: 27c,72] Supported/template/confined synthesis methods were used to obtain CDs with good monodispersity and controlled size, which specifically include soft template method[qv: 34b,73] and hard template methods.^74^ Kwon et al. synthesized oleylamine‐capped CDs by carbonization of polyacrylamide in oleylamine emulsion micelles (Figure 7e). The sizes of the CDs were tuned by changing the polyacrylamide molecular weight, which resulted in the fluorescence red‐shift as the size increased.[qv: 73a] Besides, the zeolites, silica spheres, metal–organic frameworks (MOFs), and calcium carbonate microparticles can be used as hard template to synthesize CDs.[qv: 45a,46a,74,75] Liu et al. developed a facile “dots‐in‐zeolites” strategy to confinedly synthesize CDs within a series of zeolitic crystalline matrices under solvothermal/hydrothermal conditions (Figure 7g), which resulted in CDs@zeolites composite with thermally activated delayed fluorescence (TADF).[qv: 75b] In addition to aforementioned bottom‐up methods, there are also some other methods reported to synthesize CDs. Peng and Travas‐Sejdic utilized concentrated sulfuric acid to make the carbohydrates dehydrated to produce carbonaceous materials, and further treated to obtain luminescent CDs (Figure 7f).[qv: 38a] Fan et al. regulated the chemical vapor deposition (CVD) parameters to achieve the CVD synthesis of GQDs. For the formation of GQDs, the nucleation rate and growth rate of CVD were very important. As is shown in Figure 7h, only when the nucleation rate and growth rate were within appropriate range, the GQDs could be obtained. And the sizes can be tuned by changing the nucleation rate and growth rate.^76^ The C60 molecules were also transferred into GQDs by the Ru‐catalyzed cage‐opening reaction.^77^

## 5 Synthesis of CPDs by Bottom‐Up Methods

In the last chapter, various synthesis methods of CDs have been demonstrated. Usually, the top‐down methods are breaking up the large carbon structure materials (e.g., carbon nanotube, graphite power and graphite sheets, etc.) to form carbon nanoparticles, which could be understood as teeny fragments. The obtained CDs generally partly retained the graphite structure of raw materials and possessed some groups on the surface, which can't be seen as polymer chains. So, the obtained CDs lack obvious polymer structure and properties; usually, the GQDs, CQDs, and CNDs are reached. However, even CQDs and CNDs are usually reached by the top‐down methods; the postdecoration by polymer or organic molecules can transform them into CPDs with the unambiguous carbon core and polymer shell.[qv: 33a,38] As we have known, the top‐down methods were discovered earlier than bottom‐up strategy, but the operation was generally more complicated than bottom‐up methods. Thus, CDs, which are prepared by the bottom‐up methods from small molecules or polymer precursors undergoing dehydration and further carbonization, usually reserve polymeric structure on the surface even inside the core owing to the incomplete carbonization. As a result, the CPDs are generally reached by most of bottom‐up routes, such as the hydrothermal, solvothermal, and microwave‐assistant hydrothermal/solvothermal, etc.[qv: 7b,13c,22,25a,d,e,46b,69a,78] In this section, we don't differentiate these methods and instead analyze comprehensively these synthesis methods with emphasis on the synthesis of CPDs with detailed discussion on the effect of synthesis condition.

### 5.1 The Effects of Precursors

#### 5.1.1 The Effect of Precursors' Molecular Structure

**Figure 8:**
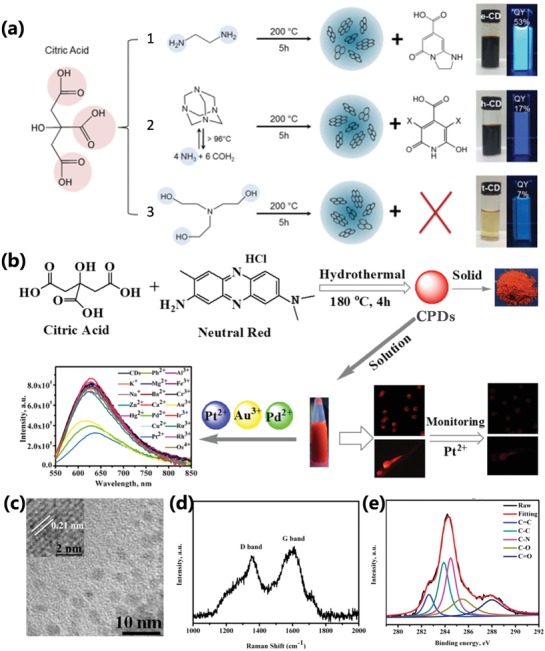
a) Schematic diagram of synthesis process of citric acid–based CPDs using three different nitrogen‐containing precursors. The molecular state produced in reaction 1 (IPCA reported in previous work) and reaction 2 (citrazinic acid and/or 3,5 derivatives (marked by −X)) contribute to the bright luminescence. In reaction 3, there isn't molecular state produced resulting in low PLQY. a) Reproduced with permission.[qv: 47a] Copyright 2016, American Chemical Society. b) Schematic diagram of synthesis route of CPDs prepared from citric acid and neutral red by hydrothermal method and applications in ions detection, bioimaging, and biosensing. c) TEM and HRTEM (inset) images of the CPDs. d) Raman spectrum of the CPDs. e) The high‐resolution XPS C1s spectra of the CPDs. b–e) Reproduced with permission.[qv: 79a] Copyright 2017, American Chemical Society.

Several kinds of precursors, including natural products (such as grass, strawberry powders, egg white), polymer, and a lot of small molecules, etc., have been used to synthesize CPDs by bottom‐up method.[qv: 7b,9c,d,25c,27b,35,47a,67,68] The precursor molecules have great effect on the properties of the CPDs in terms of the fluorescence intensity enhancing and the photoluminescent colors regulation, etc. As a result, researchers have been always trying to obtain CPDs with expectant performance by trying different precursors, but lacking of design of experiments. It can be attributed to the formation of different structures during polycondensation and carbonization process of precursors, including different conjugation domains and graphitization in carbon core, functionalization degree on the surface, specific molecular structures from polymerization of small molecules, and degree of crosslinking related with CEE effect, etc.[qv: 26a,47a,79] The CPDs prepared from CA and 1,2‐ethylenediamine (EDA) with ≈80% PLQY further proved that the strong photoluminescence originated from the molecular state called IPCA, which is formed from the condensation and inner cyclization of a citric acid and an ethylenediamine and contributes to the main blue emission.[qv: 7b,22,80] When the precursors are changed, the photoluminescence properties also have corresponding variation due to the changing of molecular state. Schneider et al. used citric acid and three different nitrogen‐containing precursors to obtain different CPDs. The CPDs from the citric acid and hexamethylenetetramine contain the molecular state of citrazinic acid and/or 3,5 derivatives. There are not molecular states existing in the CPDs prepared from the citric acid and triethanolamine. The effect of molecular state on the optical properties of CPDs was further investigated (**Figure** **8**a).[qv: 47a] The CPDs derived from citric acid and urea have also been proved exiting the molecular state emission from 4‐hydroxy‐1*H*‐pyrrolo[3,4‐c]pyridine‐1,3,6 (2*H*,5*H*)‐trione (HPPT) molecules.^43^ Besides, the precursors with aromatic structure were widely used, because they intend to form big conjugated structure with narrow bandgap, thus obtaining the long wavelength emissive CPDs (Figure 8b,c).[qv: 79a,81] Although the conjugated aromatic structure increases the rigidity, there still exist amorphous structure and a lot of surface groups for the CPDs (Figure 8d,e), which contribute to good water solubility and predominance for sensing and bioimaging even multiple functionalized applications (Figure 8b). These precursors also tend to form GQDs/CQDs through undergoing a highly carbonization and graphitization.[qv: 12c,79b] For example, Wang et al. synthesized red emissive CQDs with 53% PLQY by solvothermal condensation carbonization and dehydrogenative planarization of 1,3‐dihydroxynaphthalene and KIO_4_. The as‐prepared CQDs showed apparent graphite crystal lattice, and a strong G‐band arise in the Raman spectrum, which also indicated a good graphite structure existing in the CQDs. Besides, due to the high conjugation and high quality of good graphite structure, the high resolution C1s X‐ray photoelectron spectroscopy (XPS) spectrum showed that there are only a small number of C—OH existing except C=C structure.[qv: 79b]

**Figure 9:**
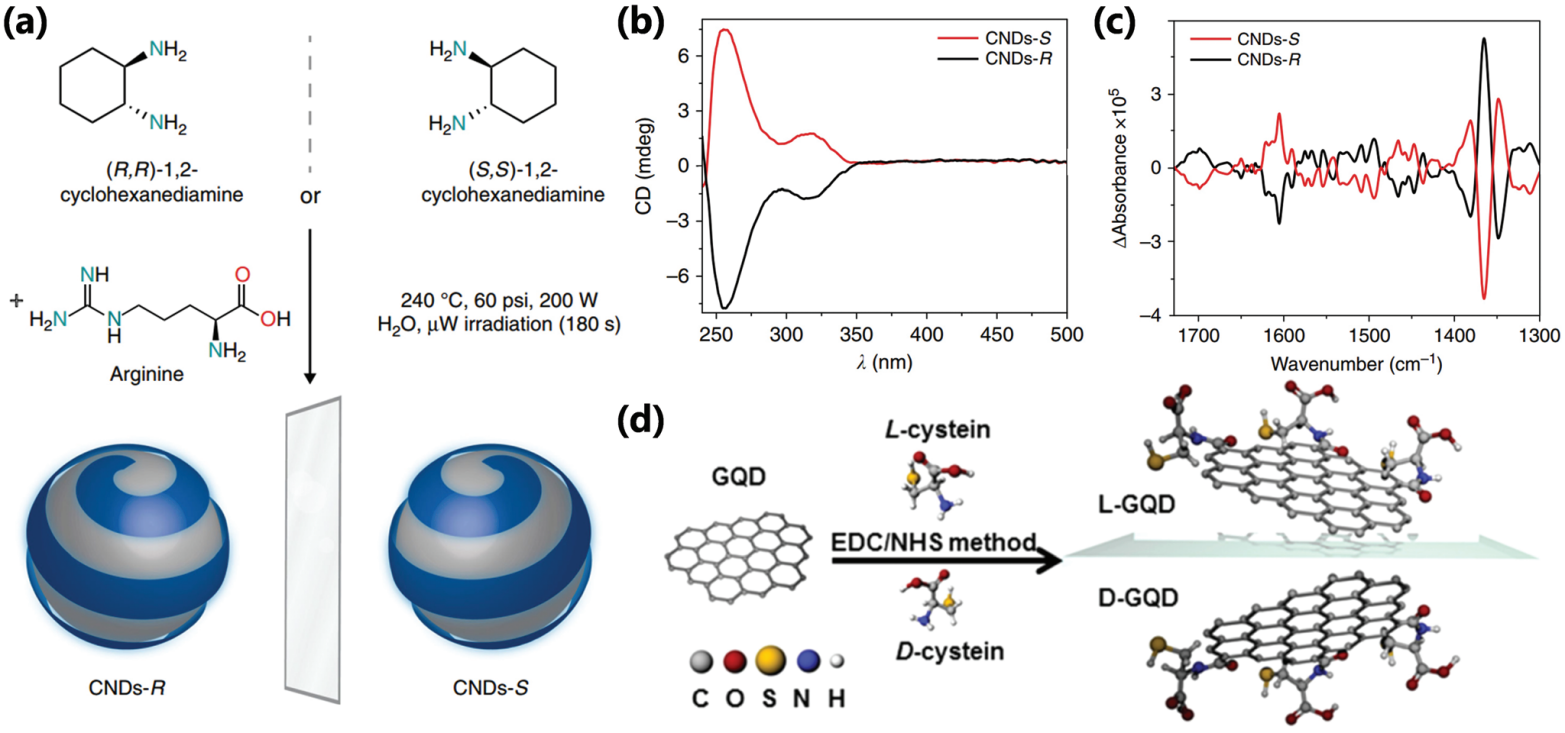
a) Schematic diagram of design and synthesis of chiral CPDs from (R,R)‐ or (S,S)‐1,2‐cyclohexanediamine and arginine by hydrothermal microwave‐assisted treatment. b) ECD spectra of CPDs‐S (red line) and CPDs‐R (black line). c) Experimental VCD spectra in water at 298 K of CPDs‐S (red line) and CPDs‐R (black line). a–c) Reproduced with permission.[qv: 82e] Copyright 2018, Nature Publishing Group. d) Schematic diagram of chiral GQDs prepared by modifying GQDs with L(D)‐cystein. d) Reproduced with permission.[qv: 42b] Copyright 2016, American Chemical Society.

Much less is known about the precise relationship between precursor molecules and special properties of CPDs. Investigating these factors is likely essential to develop novel CPDs with widely accessible features. More recently, because of the important value of chiral structure in biology and self‐assembly, the chiral properties of the CPDs have been focused in recent studies.^82^ The chiral properties of CPDs can be inherited from the precursor molecules to provide a simple strategy for the synthesis of chiral nanomaterials. Ðorđević et al. synthesized the chiral CPDs by using arginine and (R,R)‐ or (S,S)‐1,2‐cyclohexanediamine as shown in **Figure** **9**a. The electronic circular dichroism (ECD) spectroscopy of CPDs‐R and CPDs‐S respectively presented negative Cotton and positive Cotton effects, which confirmed that the chirality successfully conveys from (R,R)‐ or (S,S)‐1,2‐cyclohexanediamine to the CPDs (Figure 9b). The vibration circular spectroscopy (VCD) of CPDs‐S and CPDs‐R showed a mirror image relationship to further confirm the chiral structure of CPDs (Figure 9c).[qv: 82e] Li et al. also prepared chiral N‐S‐doped CPDs by hydrothermal treatment of chiral cysteines, and further revealed two kinds of chiral CPDs' effects on cellular energy metabolism.[qv: 82b] Although chiral CPDs can be easily prepared from chiral precursors, the chirality is usually attributed to the chiral precursor molecules retained on the surface of CPDs. However, there are still a lot of problems to explore the true origin and generation of CPDs' chirality. In fact, the chiral properties of semiconductor nanoparticles have been extensively studied by scientists, and their chiral sources are generally attributed to four aspects: asymmetry of the core of semiconductor nanoparticles; asymmetry of the surface of the core; chirality of the shell on the core surface; chiral field effect due to polarization, etc.^83^ Suzuki et al. have revealed that the chirality of chiral GQDs originated from not only the surface chirality derived from the modified chiral molecules, but also the distortion of the flexible graphene sheets, which generated the asymmetry of the core structure (Figure 9d).[qv: 42b]

In addition, amphiphilic CPDs can be prepared from some amphiphilic molecules by inheriting the amphipathy of the precursors.^84^ Some researches have also shown that the antibacterial, antitumor, and cancer‐targeting properties of CPDs are closely related to the precursor molecules used for the synthesis of CPDs.[qv: 8b,26d,85] Liu et al. used metronidazole as the sole carbon source to prepare CPDs by one‐step hydrothermal synthesis at 250 °C for 8 h. The prepared CPDs had good water solubility, nontoxicity, high brightness, and selective bacteriostatic effect for porphyromonas gingivalis. The further study shows that the selective bacteriostatic effect of CPDs can be attributed to the use of metronidazole drug molecules. The molecular structure with nitro group plays an important role in selective antibacterial effect of the obtained CPDs.[qv: 8b] Li et al. synthesized porphyrin‐containing CPDs by using mono‐hydroxylphenyl triphenylporphyrin and chitosan as the precursors. Due to the existence of porphyrin structure, the CPDs are endowed with superior photodynamic activity for antitumor therapy.[qv: 85b] Folic acid and tris(hydroxymethyl) aminomethane were also used to prepare CPDs, which have a good targeting function for cancer cells with greater advantage in water solubility, PLQY, and light stability than folic acid molecules.[qv: 26d]

#### 5.1.2 Doping

The doping strategy has been widely used for the property regulation (optical properties, catalytic performance, etc.) of the CPDs by changing the chemical and electronic structure. The common doping elements include nitrogen,[qv: 79c,86] phosphorus,^87^ sulfur,^88^ boron,[qv: 87d,89] and some metals,[qv: 26c,90] etc. Some recently reported doped CPDs are shown in **Table** **1** with the synthesis methods and some properties of CPDs. Besides, because single‐element doping is limited for the single property‐regulation, the codoping strategy is also widely adopted to regulate synergistically the properties and make the CPDs multifunctional.^39^, ^51^, ^91^

**Table 1:** Comparisons of doped atom and properties of the CPDs derived from kinds of raw materials

Main raw materials	Doped atom	Synthesis method	Main photoluminescent color	Size of CPDs [nm]	PLQY [%]	Ref.
Citric acid and ethylenediamine	Nitrogen	Hydrothermal	Blue	2–6	80	[qv: 7b]
Citric acid and urea	Nitrogen	Microwave‐assistant method	Green	1–5	14	[qv: 71a]
Citric acid and urea	Nitrogen	Solvothermal	Orange	4–10	46	64
Urea and p‐phenylenediamine	Nitrogen	Hydrothermal	Blue, green, yellow, red	about 2.6	8–35	[qv: 36c]
Dopamine and o‐phenylenediamine	Nitrogen	Hydrothermal	Red	about 7.8	26.28	[qv: 25e]
Citric acid and formamide	Nitrogen	Solvothermal	Blue, green, red	about 6.8	11.9, 16.7, 26.2	[qv: 65b]
Sucrose and orthophosphoric acid	Phosphorus	Microwave‐assistant method	Green	3–10	–	[qv: 87a]
Phosphorous tribromide and hydroquinone	Phosphorus	Solvothermal	Blue	5–15	25	[qv: 87b]
m‐Phenylenediamine, ethylenediamine and orthophosphoric acid	Nitrogen Phosphorus	Hydrothermal	Blue, green	8.1 ± 2.7	51, 38	39
DL‐malic acid and ethane sulfonic acid	Sulfur	Microwave‐assistant method	Blue	4.37 ± 0.25	5.51	[qv: 88c]
Polythiophene phenylpropionic acid	Sulfur	Hydrothermal	Red	10 ± 4	2.3	[qv: 88g]
Sodium citrate and sodium thiosulfate	Sulfur	Hydrothermal	Blue	about 4.6	67	[qv: 88d]
2,2′‐(ethylenedithio)diacetic acid	Sulfur	Soft template method	Blue, green	3.29 ± 0.45	6.48	[qv: 88f]
Boron tribromide and hydroquinone	Boron Bromine	Solvothermal	Blue	8–22	14.8	[qv: 89a]
Boric acid and sucrose	Boron	Hydrothermal	Blue	about 3.7, 5, and 6	0.5, 2.2, 2.7	[qv: 89c]
p‐Phenylenediamine and gadolinium nitrate	Nitrogen Gadolinium	Solvothermal	Orange	about 5	10.2	92
Manganese(II) phthalocyanine	Nitrogen Manganese	Solvothermal	Red	about 4.2	–	[qv: 90b]

**Figure 10:**
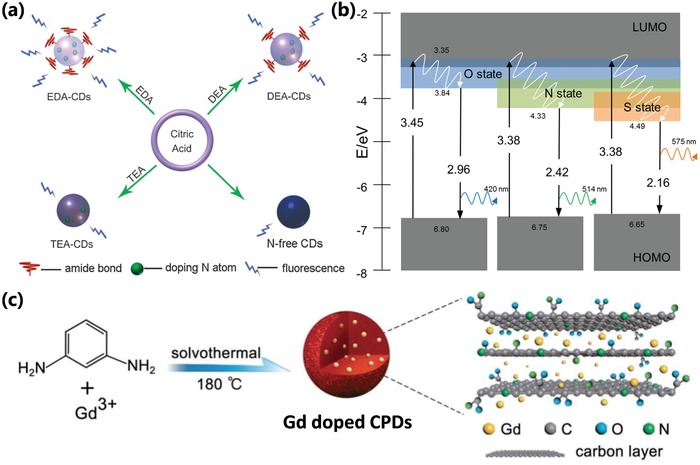
a) Schematic representation for synthesis of CPDs in the presence of various nitrogen‐containing precursors: 1,2‐ethylenediamine (EDA), diethylamine (DEA), and triethylamine (TEA). The precursors with higher content of nitrogen possess higher PLQY. a) Reproduced with permission.[qv: 79c] Copyright 2012, The Royal Society of Chemistry. b) Schematic representation of the energy level structure of CPDs with nondoping, nitrogen doping, and sulfur doping. The heteroatoms doping resulted in the variation of electronic structure and energy level. b) Reproduced with permission.[qv: 88f] Copyright 2015, Wiley‐VCH. c) Schematic illustration of the Gd‐doped CPDs prepared by solvothermal of m‐phenylenediamine and Gd(NO_3_)_3_. c) Reproduced with permission.^92^ Copyright 2018, Wiley‐VCH.

The nitrogen doping has been very universal owing to the N‐type structure and matched size with carbon atom. The nitrogen‐containing materials are also very abundant, and some nitrogenous precursors were frequently used as well‐known materials in bottom‐up method, such as ethylenediamine,[qv: 7b,25a,46b,47b] urea,[qv: 33b,64,71a] and phenylenediamine,[qv: 7a,10a,27d,69b] etc. It was generally accepted that the nitrogen doping can enhance the photoluminescence of the CPDs, and the photoluminescence wavelength can also be tailored by nitrogen doping (**Figure** **10**a). The sulfur doping CPDs were synthesized by utilizing the sulfur‐containing materials, such as sulfuric acid,[qv: 88a,91c] sodium hydrosulfide,[qv: 88b] ethane‐sulfonic acid,[qv: 88c] sodium thiosulfate,[qv: 88d] thiomalic acid,[qv: 88e] 2,2′‐(ethylenedithio)diacetic acid,[qv: 88f] etc. The doped sulfuric atoms in the carbon cluster can change the electronic structure and energy level due to the different electronegativity and lone pair of electron, resulting in the stronger photoluminescence and shift of emissive wavelength (Figure 10b).[qv: 88b,f] Similarly, the phosphorus doping and boron doping were also utilized to regulate the photoluminescence by using the phosphorus‐containing (such as phosphoric acid,[qv: 87a] phosphorous tribromide,[qv: 87b] triphenylphosphine,[qv: 87d] etc.) and boron‐containing (boric acid,[qv: 87d,89b,c] boron tribromide,[qv: 89a] etc.) raw materials. In addition to the nonmetallic element doping, the metal element doping was also frequently used to regulate CPDs' property and confer CPDs' function.[qv: 26c,90,91b,92] Chen et al. synthesized Gd‐doping CPDs from Gd(NO_3_)_3_ and m‐phenylenediamine by ethanol solvothermal (Figure 10c). The Gd‐doping CPDs were used for fluorescence imaging and magnetic resonance imaging (MRI) in vivo, and possessed good photodynamic therapy (PDT) effect with high singlet oxygen (^1^O_2_) generation under photoirradiation.^92^

### 5.2 The Effects of the Reaction Conditions

#### 5.2.1 Synthesis Temperature and Reaction Time

**Figure 11:**
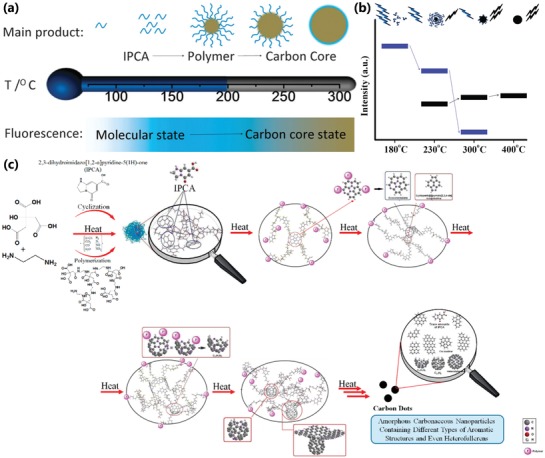
a) Schematic representation of CPDs prepared from different hydrothermal temperatures in the one‐pot hydrothermal system of CA and EDA and varying from molecular state to carbon core state. a) Reproduced with permission.^22^ Copyright 2015, The Royal Society of Chemistry. b) Schematic representation of the emission features of the CPDs from the thermal treatment of mixture of CA and EA. The molecular state (blue groups) are transformed into the carbon core state (black sphere) so that the photoluminescence contribution of molecular state (blue bars) decreases and the photoluminescence contribution of carbon core state (black bars) increases. b) Reproduced with permission.[qv: 50b] Copyright 2011, American Chemical Society. c) Schematic representation of the formation of CPDs and the variation of photoluminescent centers by pyrolyzing CA and EDA with different carbonization degree. c) Reproduced with permission.[qv: 32a] Copyright 2018, American Chemical Society.

As we discussed previously, the carbonization is very important for the formation and properties of the CPDs.[qv: 8b,13c,21e,22,26a,32a] Usually, the carbonization degree increases with the thermal treatment temperature increasing. The increasing carbonization degree in CPDs can progressively transform the molecular state to carbon core state,[qv: 22,50b] which resulted in the photoluminescent contribution of molecular state decrease and the photoluminescent contribution of carbon core state increase. So, the photoluminescence features would vary owing to the change of photoluminescent origins (**Figure** **11**a,b). Besides, the higher temperature also contributes to a higher carbonization degree and more effective graphitization, resulting in larger conjugation structure, which includes subdomain with bigger size and molecular state–related structure with a greater degree of conjugation, etc. So, the bandgap can become narrower with possible red‐shift emission. In a word, carbonization temperature could lead to the shift of emission wavelength and the variation of photoluminescence intensity. For example, the highly photoluminescence IPCA molecular state transforms into highly carbonized carbon core state at higher synthesis temperature, resulting in decreased PLQY (Figure 11a).[qv: 7b,22] Shamsipur et al. systematically analyzed the possible formation mechanism of CPDs from citric acid and ethylenediamine, as well as the change of photoluminescence centers under different carbonization degrees. At low carbonization levels, fluorescence mainly comes from molecular state (IPCA). Further carbonation resulted in the formation of larger conjugated structures in polymer/carbon hybrid structures, which contribute to the fluorescence of CPDs as conjugated subdomain state. And the further analysis shows the conjugated subdomain state can be not only the planar conjugated structure, but also the curved polycyclic aromatic hydrocarbons structure, which was experimentally supported by the mass spectrum and absorbance spectrum. Finally, the carbonization results in the formation of CNDs or CQDs with surface state or carbon core state luminescence (Figure 11c).[qv: 32a] Miao et al. synthesized CPDs with multiple color emission and obvious graphite structure by heating citric acid and urea with different molar ratio in DMF under different temperature to regulate the graphitization and surface functionalization. The results showed that higher temperature really contributes to a higher carbonization degree and graphitization, resulting in larger size and more effective conjugation structure with red‐shift emission.[qv: 33b]

**Figure 12:**
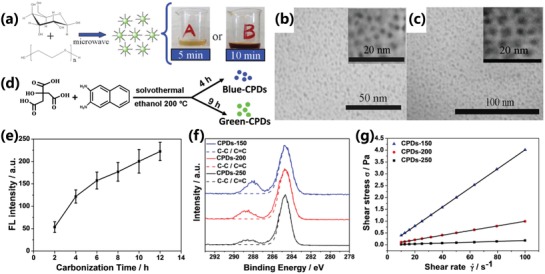
a) Schematic diagram of CPDs prepared by microwave pyrolysis approach with different reaction time. TEM images of CPDs prepared by different reaction time. (Sample A, b): the reaction time is 5 min; Sample B, c): the reaction time is 10 min). a–c) Reproduced with permission.^93^ Copyright 2009, The Royal Society of Chemistry. d) Schematic diagram of different emission CPDs prepared with different carbonization time. d) Reproduced with permission.[qv: 26a] Copyright 2016, Wiley‐VCH. e) FL intensity of same concentration CPD solution with different carbonization times. f) The high‐resolution C1s XPS spectra and the corresponding C‐C/C=C peaks of the CPDs‐150, CPDs‐200, and CPDs‐250. g) The shear stress variation with the shear rate of CPDs‐150, CPDs‐200, and CPDs‐250 solution (concentration: 50 mg mL^−1^; the slope represents the viscosity). e–g) Reproduced with permission.[qv: 13c] Copyright 2018, Wiley‐VCH.

Similarly, the longer reaction time can also lead to higher carbonization degree.[qv: 13c,26a,93] Zhu et al. synthesized CPDs utilizing poly(ethylene glycol) (PEG‐200) and saccharide as precursors by facile microwave pyrolysis approach (**Figure** **12**a). The different carbonization time resulted in CPDs with different carbonization degree and photoluminescence properties. The CPDs prepared by longer carbonization time have a higher contrast displayed in the TEM images (Figure 12b,c), which indicated that carbon core state and larger conjugated structure have a bigger contribution resulting in the red‐shift of photoluminescence.^93^ The photoluminescence wavelengths are also tunable by changing the carbonization time for the CPDs with conjugated carbon core structure similar to CQDs. For example, Yuan et al. realized the red‐shift of photoluminescence of CPDs by prolonging the carbonization time. The prolonged growth time promoted the bigger conjugated structure, resulting in the narrower bandgap (Figure 12d).[qv: 26a]

Despite discovering in crosslinking polymer systems, the CEE effect was also relevant with carbonization degree. The CEE effect shows that the crosslinking can provide a fixation effect, which can restrict the vibration and rotation to decrease the energy loss resulting in faster radiative transition rate and stronger photoluminescence. So, the increased carbonization degree can result in stronger photoluminescence within a certain range owing to the increasing of chemical crosslinking of groups and physical crosslinking of the rigid carbon structure, which is regarded as carbonization‐induced CEE effect.[qv: 8b,13c,45b] Xia et al. synthesized CPDs by different carbonization time and carbonization temperature. The CPDs synthesized by longer carbonization time possessed a stronger photoluminescence emission (Figure 12e). Similarly, the higher carbonization temperature also resulted in stronger photoluminescence. The PLQY of the CPDs‐150, CPDs‐200, and CPDs‐250 prepared in 150, 200, and 250 °C were 10.20%, 13.88%, and 25.57%, respectively. The XPS analysis showed the content of C—C/C=C increased as carbonization temperatures elevated (Figure 12f), which revealed the carbonization degree and graphitization degree really increased. Besides, the decreased viscosity (Figure 12g), as carbonization degree increased, showed the polymer property weakened, which would lead to the enhancement of restriction.[qv: 13c]

#### 5.2.2 pH of the Solution

**Figure 13:**
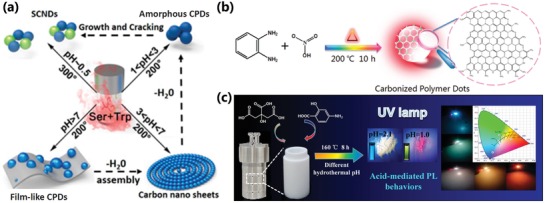
a) Schematic representation of the preparation procedure of supersmall CNDs (SCNDs), amorphous CPDs, film‐like CPDs, and carbon nanosheets by hydrothermal carbonization of L‐serine and L‐tryptophan at different PH values and temperatures. a) Reproduced with permission.^94^ Copyright 2016, American Chemical Society. b) The possible formation mechanism of red emissive CPDs with conjugated aromatic benzene skeleton in the presence of HNO_3_ under high temperature and high pressure. b) Reproduced with permission.[qv: 10a] Copyright 2018, Wiley‐VCH. c) Schematic illustration of the preparation and LED application of acid‐mediated photoluminescent CPDs with solid state and solution emission. c) Reproduced with permission.^81^ Copyright 2017, American Chemical Society.

As we all know, for the polymerization reaction, the pH always plays an important role. Similarly, for the polymerization and carbonization process of CPDs, the pH of the hydrothermal mixed solution has a great impact on the properties of the finally obtained CPDs owing to catalysis effect and the variety of the existence form of the functional groups, such as protonation.[qv: 10a,16b,25e,81,94] The pH‐dependent synthesis of novel structure‐controllable CPDs was reported firstly by Lu et al.^94^ The film‐like CPDs, polymer carbon nanosheets, and amorphous CPDs were respectively obtained by altering the initial pH of the solution (**Figure** **13**a), and their photoluminescence properties were also enormously influenced. The changes of the morphology and the optical properties were attributed to the decreased pH that resulted in the increased carbonization degree. The reaction mechanism was speculated that the hydrogen ion can serve as a catalysis to promote the dehydration reaction.^81^, ^94^ Besides, supersmall CNDs, which possessed high carbonization degree prepared in the condition of higher temperature and lower pH, have direct white photoluminescence. So, it is easily to apply the supersmall CNDs as color transformation layer for white emission LED.^94^ Furthermore, Lu et al. synthesized near‐infrared photoluminescence CPDs by adding HCl to regulate the pH of precursor solution. The obtained CPDs possessed two‐photon fluorescence and were applied for bioimaging and red‐emission LED.[qv: 25e] Liu et al. synthesized red‐emissive CPDs by adding HNO_3_ into o‐phenylenediamine via one‐step hydrothermal treatment. In the reaction, the HNO_3_ not only acted as catalyst to accelerate the reaction rate but also affected the structure of CPDs by the nitration, reduction, protonation, oxidative polymerization, and carbonization reactions (Figure 13b).[qv: 10a] Feng et al. further realized the solid‐state and solution emission changing of the CPDs by the acid‐mediated strategy, which were applied for full‐color emission LED (Figure 13c).^81^ Yuan et al. also utilized the acid to achieve the accelerated carbonization and growth of CQDs, which resulted in bigger sizes with red‐shift emission due to quantum size effect.[qv: 16b]

### 5.3 The Nucleation Mechanism and Formation Process of CPDs

Although a lot of CPDs have been prepared from various precursors, the nucleation mechanism and forming process of CPDs have been rarely investigated. Usually, the CPDs were synthesized by hydrothermal/solvothermal treatment in a sealed reactor, so the forming process of CPDs is difficult to investigate in a like‐black box reaction. Besides, the use of various precursors and the complicated chemical reaction under high temperature and high pressure make it more difficult to reach the universal principle to reveal the nucleation mechanism and make the formation process of CPDs controllable.[qv: 7b,13c,20]

**Figure 14:**
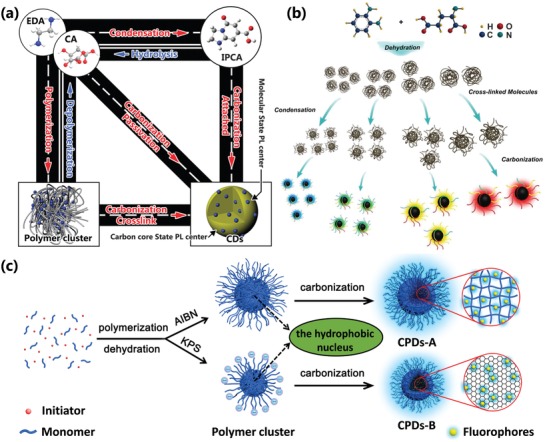
a) The scheme of the conversion process between different products for synthesis of the CPDs in the one‐pot hydrothermal system of CA and EDA. a) Reproduced with permission.^22^ Copyright 2015, The Royal Society of Chemistry. b) The scheme of formation mechanism of CPDs with tunable photoluminescence from condensation crosslinking and carbonization of L‐glutamic acid and o‐phenylenediamine by solvothermal. b) Reproduced with permission.[qv: 27d] Copyright 2018, Wiley‐VCH. c) The schematic diagram of the nucleation and reaction process of the CPDs from hydrothermal addition polymerization and carbonization of acrylamide monomers triggered by initiator. c) Reproduced with permission.[qv: 13c] Copyright 2018, Wiley‐VCH.

The CPDs are formed from the carbonization of polymer clusters is universally accepted.[qv: 13c,20,21c,22,32a,95] The small molecules and/or polymer precursors with a lot of amine, carboxyl, hydroxyl first react with each other by polycondensation to form small crosslinked polymer clusters, and further undergo crosslinking and carbonization resulting in the formation of CPDs (**Figure** **14**a,b).[qv: 22,27d,32a] The conditions of dehydration and carbonization have large influences on the sizes and graphitization degree of CPDs, which results in the variation of photoluminescence properties (Figure 14b). The polymers are also used to synthesize CPDs in the solution, the tangled polymer cluster transfer into CPDs undergoing dehydration and carbonization. The carbonization is crucial for forming CPDs; the polymers without fluorescence in nature were carbonized by hydrothermal to reach highly photoluminescent CPDs.[qv: 25c,27b]

**Figure 15:**
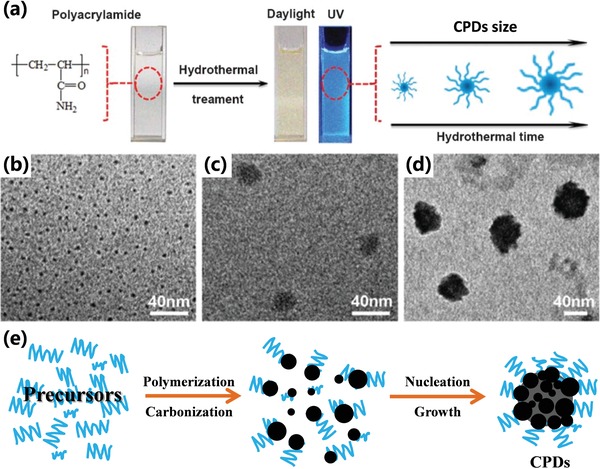
a) Schematic illustration of the formation of CPDs by hydrothermal treatment of polyacrylamide and the digital optical photos of CPD aqueous solution under daylight and UV irradiation. The CPDs grow larger with longer hydrothermal time. The TEM images of CPDs prepared by b) 24 h hydrothermal time, c) 72 h hydrothermal time, and d) 96 h hydrothermal time. a–d) Reproduced with permission.[qv: 27b] Copyright 2013, The Royal Society of Chemistry. e) Schematic illustration of formation mechanism of CPDs from small molecule and/or polymer precursors by polymerization and carbonization.

However, the nucleation mechanism is still unclear. In recent work, Xia et al. assumed the nucleation of CPDs was similar to the soap‐free emulsion polymerization.[qv: 13c] To begin with, the polymerization resulted in the formation of polymer clusters. After crosslinking and dehydration, the hydrophobicity of the polymer clusters increased, which led to hydrophobic structure inside and hydrophilic structure outside resulting in the microphase separation to form the nucleus (Figure 14c). Qu and co‐workers also utilize the hydrophobicity–hydrophilicity interaction to prepare the supra‐CDs as nanobombs, which was similar with the formation of micelle.[qv: 13a] After the nucleation, the small molecules or polymer in the solution will further attach to the surface of the nucleus and the nucleus further dehydrates and carbonizes to form a highly crosslinked structure or carbon skeleton with graphite or like‐diamond structure, the formation of the neosurface and the enlargement of the carbon core result in growth of the CPDs. So, the longer reaction time would result in bigger CPDs (**Figure** **15**a–d).[qv: 27b,f,89c] When the reaction of the small molecules or polymer in the solution and the surface functional groups of the CPDs reach to equilibrium, the growth will terminate. It also shows that the reverse reaction would also restrain the precursors transform CPDs.[qv: 13c,22] It should be noted that the forming of the crosslinked structure would enhance the hydrophobicity, which leads to the decreasing of the water concentration and microphase separation in the nuclear area, resulting in easier to dehydrate and carbonize, so the dehydration reaction could continuously proceed, and finally, the CPDs with a highly crosslinked structure or a highly carbonization core are obtained. It is the reason why the CPDs can be obtained under some hydrolytic reactions rather than decompose into small molecules.^20^, ^22^ Besides, CPDs with multiple subdomain might be obtained by the aggregation of several micronucleus, which are formed in the initial stage. It results in that a bigger nucleus forms and further grows and carbonizes to reach CPDs with multiple subdomains. In fact, the aggregation of the micronucleus could be conducted by the hydrophilic and hydrophobility effect, which is similar to the formation of micelle, where the hydrophobility part accumulates inside and the hydrophilic part wraps outside (Figure 15e).[qv: 27c,34a] Similarly, in the initial stage, if the polymer clusters tend to aggregate and form bigger and stable polymer cluster, the obtained CPDs would possess bigger size. On the contrary, if it is difficult to aggregate, only the smaller size CPDs would be obtained. For example, the polymer cluster with abundant surface charge wouldn't aggregate to form bigger polymer cluster due to the charge repulsive force, resulting in small CPDs.[qv: 13c]

## 6 Conclusions and Perspective

CDs, as an excellent fluorescent carbon nanomaterial, have been widely studied since it was first reported in 2004. In more than a decade, a large number of CDs have been synthesized through kinds of synthesis methods with great differences in structure and performance characteristics. In this review, according to the structure and property features, the new classification of CDs was put forward including GQDs, CQDs, CNDs, and CPDs, which would be beneficial for bringing light to the essence of CDs. Especially, it is revealed the CPD as a new kind of CD is emerging, possessing distinctive polymer/carbon hybrid structure and property. Further, we summarized various synthesis methods for preparation of CDs to reveal the effect of synthesis on structure and property features of CDs, especially focusing on the bottom‐up methods, in which the CPDs were mainly obtained through incomplete carbonization of polymer clusters. The effects of synthesis conditions on the properties and structures of CPDs were analyzed. It is significant to establish the relationship between the synthesis and the structures/properties. Furthermore, the nucleation mechanism of the formation process of CPDs was discussed and analyzed according to the previous reports.

Although great progress has been made in the research of CPDs, the development of bottom‐up synthesis methods making a large number of CPDs with various excellent properties has been conveniently synthesized. However, in the previous researches, the researchers have always focused on synthesizing kinds of CPDs with different properties from various raw materials. We think the future researches should focus more on the principle of synthesis, and to deeply reveal the reasons how the synthesis determines the structure and performance. Up to now, the synthesis of CPDs cannot be well controlled to achieve structural and performance regulation, which extremely restricts the application of the CPDs, such as the tune of photoluminescence wavelength for LED, CPDs with targeting function for biolabeling and biotherapy, controllable surface groups for sensing, etc. The specific reaction mechanism, nucleation mechanism, and formation process are also unclear, most of the synthesis principle comes from the hypothesis, which makes it difficult to raise the yield and obtain the CPDs with controllable sizes. Even the exact structures of CPDs are still unclear because of the difficult researches on complex polymer/carbon hybrid structure, and lack of systematic structure characteristic measures to reveal this structural feature. These all require a lot of exploration with enormous research challenge and prospects. The exploration and solution of the above problems would make us realize the controlled synthesis of CPDs to make this material more valuable in the fields of biomedicine, anticounterfeiting, sensing, catalysis, light‐emitting diode, and photovoltaic devices, etc.

## Conflict of Interest

The authors declare no conflict of interest.
